# Improvement of water quality for mass anopheline rearing: evaluation of the impact of ammonia-capturing zeolite on larval development and adult phenotypic quality

**DOI:** 10.1186/s13071-021-04763-w

**Published:** 2021-05-20

**Authors:** Nwamaka Oluchukwu Akpodiete, Frdric Tripet

**Affiliations:** Centre for Applied Entomology and Parasitology, School of Life Sciences, Keele University, Staffordshire, UK

**Keywords:** Zeolite, Biological filtration, Chemical filtration, Mosquito mass rearing, Sterile insect technique SIT, Genetically modified mosquitoes (GMM), Release programmes, Sustainability, Water scarcity

## Abstract

**Background:**

Malaria vector control approaches that rely on mosquito releases such as the sterile insect technique (SIT) and suppression or replacement strategies relying on genetically modified mosquitoes (GMM) depend on effective mass production of *Anopheles* mosquitoes. Anophelines typically require relatively clean larval rearing water, and water management techniques that minimise toxic ammonia are key to achieving optimal rearing conditions in small and large rearing facilities. Zeolites are extensively used in closed-system fish aquaculture to improve water quality and reduce water consumption, thanks to their selective adsorption of ammonia and toxic heavy metals. The many advantages of zeolites include low cost, abundance in many parts of the world and environmental friendliness. However, so far, their potential benefit for mosquito rearing has not been evaluated.

**Methods:**

This study evaluated the independent effects of zeolite and daily water changes (to simulate a continuous flow system) on the rearing of *An. coluzzii* under two feed regimes (powder and slurry feed) and larval densities (200 and 400 larvae per tray). The duration of larval development, adult emergence success and phenotypic quality (body size) were recorded to assess the impact of water treatments on mosquito numbers, phenotypic quality and identification of optimal feeding regimes and larval density for the use of zeolite.

**Results:**

Overall, mosquito emergence, duration of development and adult phenotypic quality were significantly better in treatments with daily water changes. In treatments without daily water changes, zeolite significantly improved water quality at the lower larval rearing density, resulting in higher mosquito emergence and shorter development time. At the lower larval rearing density, the adult phenotypic quality did not significantly differ between zeolite treatment without water changes and those with daily changes.

**Conclusions:**

These results suggest that treating rearing water with zeolite can improve mosquito production in smaller facilities. Zeolite could also offer cost-effective and environmentally friendly solutions for water recycling management systems in larger production facilities. Further studies are needed to optimise and assess the costs and benefits of such applications to *Anopheles gambiae* (*s.l.*) mosquito-rearing programmes.

**Graphic abstract:**

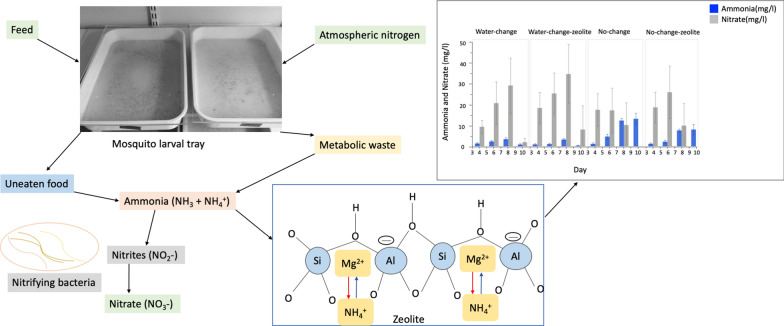

**Supplementary Information:**

The online version contains supplementary material available at 10.1186/s13071-021-04763-w.

## Background

In sub-Saharan Africa, the primary vectors of malaria are found in the *Anopheles gambiae* (*s.l.*) species complex, with members *An. gambiae* (*s.s.*), *An. coluzzii* and *An. arabiensis* transmitting malaria over vast ranges of sub-Saharan Africa and the surrounding islands [1]. Due to insecticide resistance, there is an increasing demand for complementary or novel approaches to vector control, such as the sterile insect technique (SIT), that are effective, sustainable, environmentally friendly and able to sustain the progress that has been made toward reduction and elimination of malaria transmission [2, 3].

The success of SIT and other mass-release based vector control approaches relies on large-scale production of mosquitoes and is dependent on a reliable supply of constant water of sufficient quality [4, 5]. In their natural environment, *An. gambiae* (*s.s.*), *An. coluzzii* and *An. arabiensis* typically use larval habitats with comparatively cleaner water than *Culex* and aedine species, and their larvae do not survive in water with high organic and bacterial content [6,8]. This requirement is carried over to the insectary, where mosquito larvae succumb at high ammonia levels and do not tolerate heavy bacterial growth [9]. Effective water management in mosquito insectaries that avoids waste and bacteria build-up is key to achieving optimal rearing results for small and large mass-rearing facilities [4, 9, 10]. This implies that rearing facilities rely on water replenishments and, in a few facilities, continuous-flow systems to maintain water quality whilst providing optimal diet availability [11, 12]. If only clean water were to be used for this purpose, large amounts of water would be required. For example, for SIT production centres, the FAO/IAEA recommends a larval rearing rack (holding up to 200,000 *Anopheles* larvae) using approximately 250 l per cohort [4, 10, 13]. Approximately 100,000 l of water is required to produce 10,000,000 sterile males per week [4, 10]. In an attempt to reduce water consumption, the FAO/IAEA research laboratory has also tested reusing larval rearing water treated by ultrafiltration (UF) and reverse osmosis (RO) for successive mosquito generations [4, 9]. Whilst the results showed success in rearing outcomes, there are other water treatment techniques involving mechanical, chemical and biological filtration that are currently in use for recycling water in fish aquaculture but remain to be evaluated for mosquito rearing [4, 9].

Zeolites are microporous crystalline aluminosilicates with chemically neutral basic honeycomb-like structures [14]. This chemical structure of zeolite forms a network of channels and cavities, allowing easy penetration of molecules that are filtered according to size, polarity and shape, thereby serving as an efficient filter that absorbs various substances such as ammonia, heavy metals, pesticides, odours, radioactive cations and many other toxins [15]. Zeolites have an excellent ion exchange capability for cations and prefer those with greater radius and monovalent charge, hence their affinity for cations such as ammonium ion (NH_4_^+^) [14, 16]. Due to their porous nature, the ion exchange occurs not only at the surface but also deep within the zeolite structure, further enhancing its adsorption efficiency [17, 18]. There are more than 60 types of naturally occurring zeolites with 150 synthetic types formulated with improved efficiency [14]. Natural zeolites are abundant in many parts of the world where thick deposition and contemporaneous volcanism occurred, such as New Zealand, Japan. Korea, Alaska, the western United States, Sakhalin, Kamchatka, Chile and other potential areas in the Tethys region [19].

These properties have caught the aquaculture industrys attention, resulting in an industry-wide application of zeolite in fish and crustacean aquaculture to improve water and feed quality, reduce the negative environmental impacts of aquaculture and improve the quality of seafood [14, 15]. In closed-system fish aquaculture research, zeolite has been integrated into biofilters to improve efficiency, for live fish transportation to prevent ammonia accumulation and as an additive to improve fish growth and health [14, 15]. A recent study showed that zeolite's use improved European seabasss survival rate by 12% and growth performance compared to control [20]. Another study showed increased feed consumption and utilisation, improved growth rate and phenotypic quality, and reduced mortality resulting in a 31% increase in economic returns when *Oreochromis niloticus* rearing water was treated with zeolite [21]. In addition, following saturation, zeolite can easily and cheaply be recharged by soaking in 10% NaCl solution and reused [14, 17, 18].

In this study, the use of zeolite treatment was evaluated by rearing the Mopti strain of *An. coluzzii* in comparison and/or combination with a continuous flow system (simulated by daily water changes). Results showed that under certain conditions, treating rearing water with zeolite could significantly improve production in small facilities. The possible use of zeolite to complement or offer a cheaper alternative to water treatment steps such as ultrafiltration, reverse osmosis or biological filtration, which are sometimes part of larger continuous water flow and water recycling systems, is discussed.

## Methods

### Mosquito strain

The Mopti strain of *An. coluzzii*, colonised in 2003 by the Lanzaro Laboratory (UC Davis) from the village of NGabacoro droit near Bamako, Mali, West Africa, was used for the experiments. The strain was maintained by the Tripet Laboratory in dedicated insectaries of the Centre of Applied Entomology and Parasitology (CAEP), Keele University, UK. Mosquitoes were maintained at 252 C, relative humidity of 705%, with a 12-h light/dark photocycle. Larvae were fed a diet of ground fish food flakes (Tetramin, Tetra, Melle, Germany) at a rearing density of 200 larvae/l [22]. Pupae were transferred to 5-l plastic cages (20.5 cm height20 cm diameter), covered with netting for adult emergence. Cages had a sleeved opening for easy management of mosquitoes and accessories. Approximately 600800 adults were held in a cage; sugar was provided via a paper towel soaked in 10% glucose solution and water via a soaked cotton pad in an upturned bowl placed on the cage netting. Female adult mosquitoes were fed with horse blood using an artificial feeding membrane (Hemotek feeding membrane system, Discovery Workshops, Blackburn, UK). Styrofoam cups (egg cups) containing filter paper and water were placed in the cages 4 days post-blood-feeding to collect eggs. Following the removal of the egg cups, the cages were washed thoroughly and sterilised with bleach. Mouth aspirators were used to transfer adults from one container to another when necessary.

### Experimental design: effect of zeolite treatment, water changes, feed regimes and larval density on the development and phenotypic quality of *An. coluzzii*

**Fig. 1:**
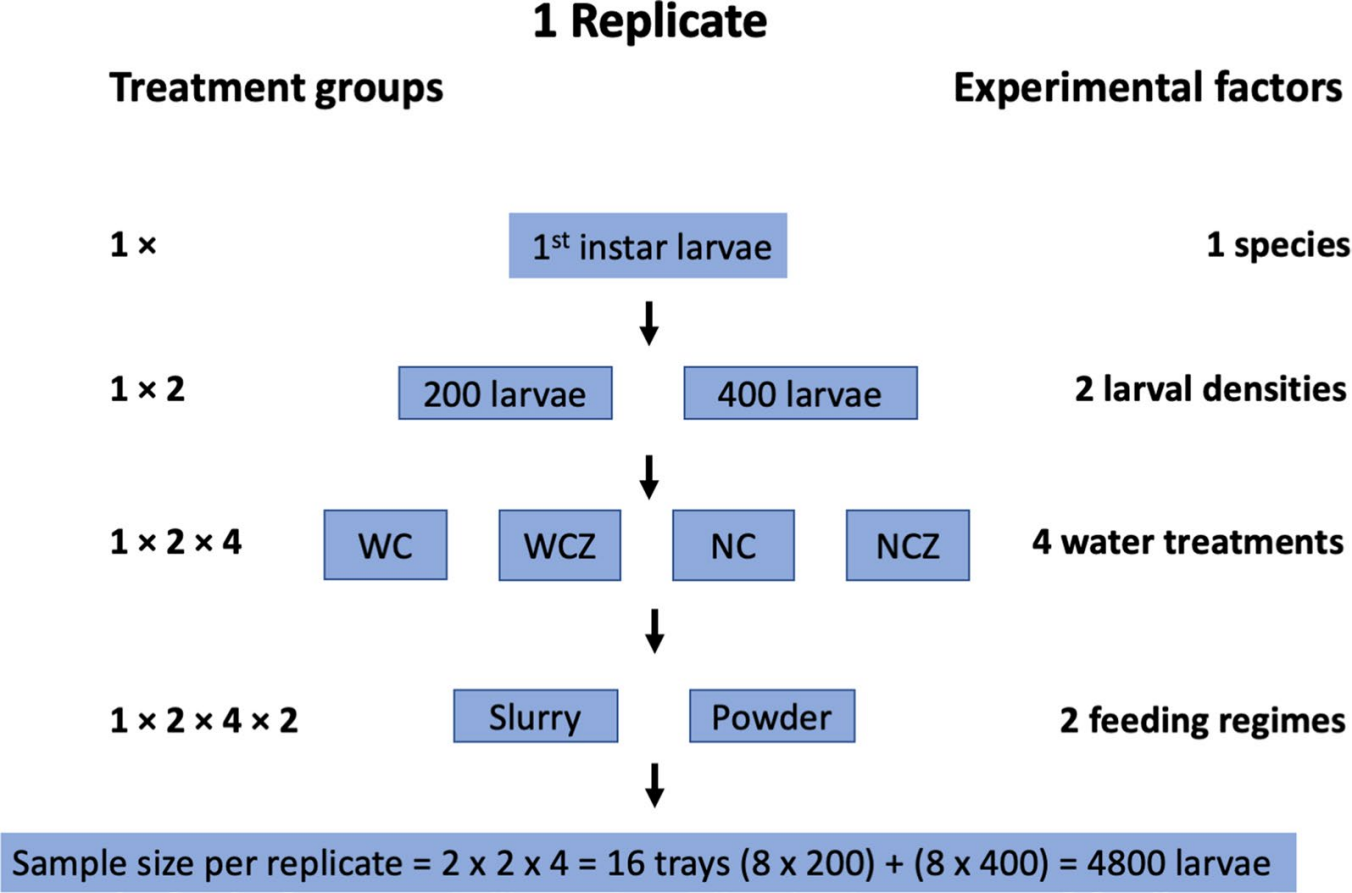
Experimental design showing experimental factors combined in one replicate resulting in two larval densities, four experimental water treatments (WC, WCZ, NC, NCZ) and two feeding regimes (powder and slurry)

First instar larvae of *An. coluzzii* were reared at two larval rearing densities (200 and 400 larvae per tray) under four different water treatments and using two different feed regimes. This resulted in a fully balanced 242 design and 16 larval trays per replicate with a total sample size of 19,200 larvae for four replicates. Trays were identified with coloured tapes codes and fully randomised in their positions on the insectary shelves (Fig. 1).

Mosquito larvae were reared in mineral water containing natural minerals sourced in 5 l bottled water from Tesco supermarket. Water quality specifications for mineral water were: TDS (112.212 mg/l), salinity (75.781 ppm) and conductivity (160.402 s). This water contained the following minerals per litre: calcium (11 mg), magnesium (3.5 mg), potassium (2.5 mg), sodium (10 mg), bicarbonate (25 mg), sulphate (11 mg), nitrate (15 mg), chloride (14 mg) and dry residue at 180 C (85 mg) and pH 6.2.

Larvae were reared in four water treatment groups: Water change (WC): first instar larvae were transferred to trays containing 500 ml of mineral water on day 1. On day 5, 400 ml of water was gently drained from the trays using a low-pressured water pump through a filter net to prevent mosquito larvae escaping into the pump, after which 900 ml of fresh mineral water was added to the tray. This process of gently draining rearing water and replacing it with fresh water was repeated daily from day 5 until all mosquitoes in the tray had pupated (Fig. 1). Water-change-zeolite (WCZ): the same set-up as in WC and 1 g of finely ground zeolite powder (Natural Clinoptilolite, Minerals-Water, Rainham, UK) was added to the rearing water on day 4 (Fig. 1). The draining process did not result in a significant loss of zeolite; water is drained gently, avoiding zeolite particles that have settled at the bottom of the tray. No-change (NC)first instar larvae were initially transferred to trays containing 500 ml of mineral water and received an additional 500 ml of mineral water on day 5 (Fig. 1). No-change-zeolite (NCZ)On day 1, first instar larvae were transferred to trays containing 500 ml of mineral water; on day 4, 1 g of finely ground zeolite powder was added to the rearing water; on day 5, 500 ml of additional mineral water was added to the rearing trays (Fig. 1).

Larvae were fed with two different standardised feeding regimes (slurry and powder feed), except on day 1, where 0.1 ml of Liquifry liquid fish food (Interpret Ltd, Surrey, UK) was used to feed first instar larvae. The powder feeding regime consists of daily rations of ground fish food, using a spatula to spread it on the water surface: 6 mg on days 23, 30 mg on day 4 and 60 mg on day 5 until pupation. The slurry feeding regime consists of the same food quantity suspended in deionised water (1 ml of 60 mg/10 ml of TetraMin Baby on days 23, 1 ml of 300 mg/10 ml of TetraMin Baby on day 4 and 1 ml of 600 mg/10 ml of TetraMin Baby on day 5 until pupation) and injected into the larval trays using a pipette. Pupae were picked from larval trays using 3ml plastic pipettes, transferred to styrofoam cups containing mineral water and then placed in adult cages for emergence (Fig. 1).

Depending on the mosquitoes life-cycle stage, the following data were observed and recorded: (i) larval survival: determined as the percentage of larvae that developed into pupae from the total number of larvae for each water treatment; (ii) pupal mortality: determined as the number of mosquitoes that died at pupation; (iii) adult emergence: determined as the percentage of mosquitoes that emerged as adults from the total number of larvae in each water treatment; (iv) development time: determined as the number of days from placement of first instar larvae in water treatment trays until adult emergence; (v) wing length: emerged adults were collected using a mouth aspirator, sexed and stored in 75% ethanol for subsequent wing-length measurement. One wing of each emerged adult was measured from the distal end of the allula to the apical margin (radius veins), excluding the fringe scale, using a binocular microscope. A 1mm stage micrometre (Graticules Ltd, Kent, UK) was used for calibration at 25 magnification on a scale of 1 microscope unit=0.04 mm [23]. A total of 1280 emerged adults equivalent to 40 males and 40 females per treatment were randomly sampled for wing-length measurements. At the end of the entire experiment, this random selection was made to account for late-emerging adults likely being larger.

### Physicochemical properties of larval trays

Measurements for ammonia (NH_3_) were taken using a Handheld Colorimeter kit (Hanna Instruments, USA), nitrate was measured using API aquarium test kits (Mars Fishcare North America, Inc, Chalfont, USA) on days 4, 6, 8 and 10 (if larvae were still alive in the tray) following experimental set-up (Additional file 1: Table S1).

### Statistical analysis

All data collected were analysed using JMP 14 (SAS Institute, Inc., Cary, NC, USA). All data were checked for deviations from normality and heterogeneity of variance, and analyses were conducted using parametric and non-parametric methods as appropriate. The 242 design of the experiment allowed for fully balanced multivariate statistical models. In multivariate analyses, replicate effects were tested and only reported when significant. Interactions between independent variables were tested using a stepwise approach, and only those found to be significant were retained in the final models. For analyses of proportion of larvae, pupae and adults, likelihood odds ratios were used for *post hoc* pairwise group comparisons following logistic regressions. Body size was analysed through general linear models followed by Tukeys HSD *post hoc* pairwise comparisons. Developmental times (day of emergence) were analysed by Cox proportional hazard models with likelihood odds ratios for *post hoc* pairwise comparisons. Finally, ammonia and nitrate measurements were analysed through a generalised linear model using standard least squares. A quadratic term was added to account for non-linearity of the effect of day on ammonia concentration.

## Results

### Physicochemical properties of mosquito larval water

**Fig. 2:**
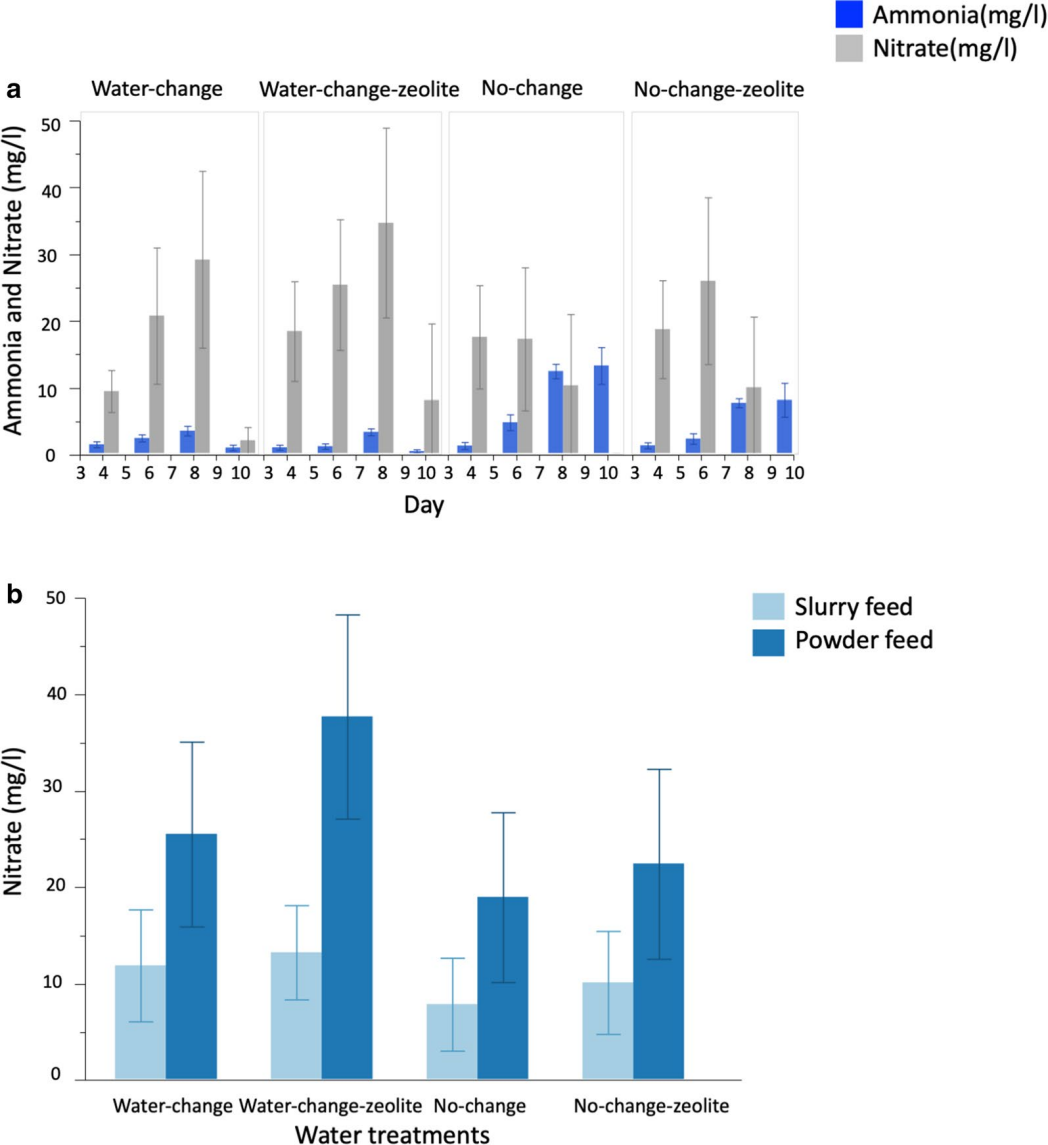
**a**, **b** Ammonia and nitrate concentration across water treatments. **a** Ammonia (blue bars) and nitrate (grey bars) concentrations across water treatments. **b** Nitrate concentration by feed regimes (slurry feed: light blue bars, powder feed: dark blue bars) across water treatments. Whiskers represent 95% confidence intervals (CI)

Overall, ammonia concentrations in mosquito larval trays were significantly impacted by water treatment (Table 1). Ammonia concentrations in the No-change (NC) and No-change-zeolite (NCZ) treatments were significantly higher than in the Daily-change (WC) and Daily-change-zeolite (WCZ) treatments (Tukey HSD test: *t*-ratio>9.96, *P*<0.0029). Ammonia concentration was significantly lower in NCZ compared to NC (*P*<0.0001) and in WCZ compared to WC (*P*=0.0029) (Fig. 2a; Additional file 1: Table S1). Day of experimentation significantly impacted ammonia concentrations in larval trays, rising markedly from day 4 (Table 1; Fig. 2a). A significant interaction between water treatment and day of experimentation also impacted ammonia concentration in mosquito larval trays (Table 1). For instance, in NC and NCZ, there was a steady build-up of ammonia from day 4 and reaching a peak on day 10. Inversely, in WC and WCZ, ammonia concentrations were relatively low and stable throughout the experiment (Fig. 2a; Additional file 1: Table S1). Feed regimes and larval rearing density did not significantly impact ammonia concentrations in larval trays (Table 1).

**Table 1:** General linear model of ammonia and nitrate concentrations across water treatments

Parameter	Source	df	*F*-ratio	*P*-value
Ammonia (mg/l)	Feed	1	0.605	0.4374^ns^
	Larval density	1	0.4077	0.5238^ns^
	Water treatment	3	88.361	<0.0001***
	Day	1	171.397	<0.0001***
	Water treatment*day	3	56.165	<0.0001***
	Day*day	1	82.230	<0.0001***
Nitrate (mg/l)	Feed	1	40.497	<0.0001***
	Larval density	1	3.167	0.0764 ns
	Water treatment	3	12.992	<0.0001***
	Day	1	128.072	<0.0001***
	Water treatment*day	3	6.202	0.0005**
	Day*day	1	61.472	<0.0001***

*P*-value: ***<0.0001 (most significant), **<0.005, *<0.05, ^ns^>0.05 (not significant)

*df* Degrees of freedom. A quadratic term (Day*Day) was added to account for non-linearity of the effect of day on ammonia concentration

Nitrate concentrations in larval trays were also significantly affected by water treatment (Table 1). Nitrate levels were significantly higher in treatments with water changes (WC and WCZ) compared to those without (NC and NCZ) (Tukey HSD tests: *t* ratios>5.62 and *P* values<0.0041 in all cases) (Fig. 2b; Additional file 1: Table S1). There was a significant increase in nitrate concentrations from day 4 to day 10 (Table 1). Nitrate levels significantly increased with time in WC and WCZ from day 4 to day 8, reducing by day 10 (Table 1). In the no-change groups (NCZ and NC), nitrate increased from day 4 to day 6, reducing by day 8 (Fig. 2b). Overall, nitrate concentrations were significantly higher in powder feed than slurry feed (Table 1; Fig. 2b). Larval rearing density did not have a significant impact on nitrate concentrations (Table 1).

### Effect of larval density, water treatment and feed regimes on larval survival

**Fig. 3:**
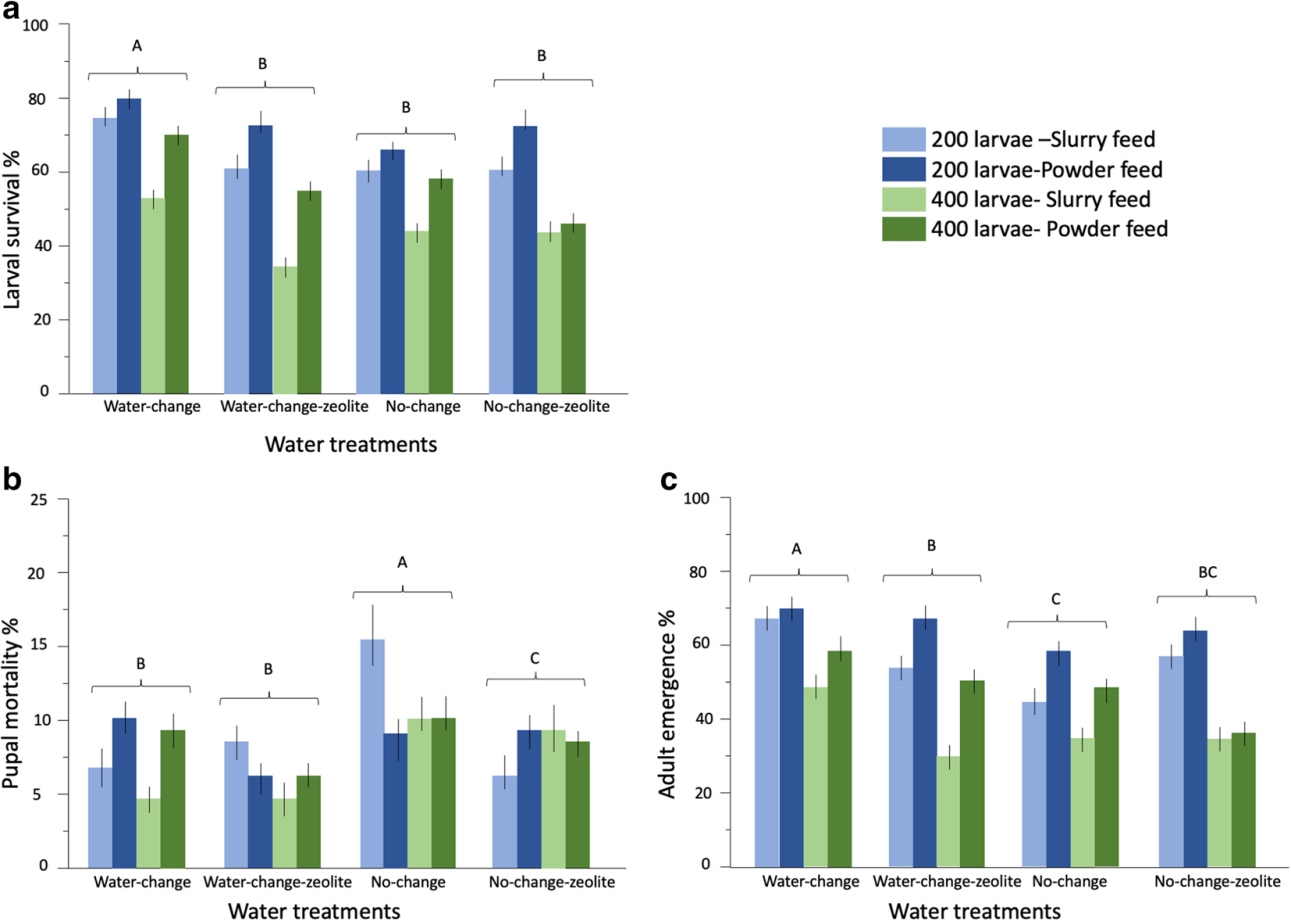
**ac** Developmental success of *An. coluzzii* across water treatments, feed regimes and larval densities. Whiskers represent 95% confidence intervals (CI)

Larval survival was significantly impacted by water treatment (Table 2). Pairwise comparisons revealed that larval survival was significantly higher in the WC (66%) in comparison to other water treatments (odds ratio test: *P*<0.0001) (Fig. 3a; Additional file 2: Table S2). Larval survival was significantly higher (10%) in powder feed than slurry feed (Fig. 3a; Table 2; Additional file 2: Table S2). Larval survival was significantly higher at 200 larval density than in 400 (Table 2). The significant interaction between water treatment and feed regime resulted in the lowest survival in NC for powder feed and WCZ for slurry feed (Table 2; Fig. 3a; Additional file 2: Table S2). A significant interaction between water treatment and density resulted in the lowest survival at 200 rearing density in NC and at 400 rearing density in WCZ (Table 2; Fig. 3a; Additional file 2: Table S2).

**Table 2:** Nominal logistic regressions of the effects of water treatments, larval density and feed regimes on mosquito survival

Parameter	Source	DF	Likelihood ratio	*P*-value
Larval survival	Larval density	1	610.267	<0.0001***
	Feed	1	195.915	<0.0001***
	Water treatment	3	221.067	<0.0001***
	Water treatment*feed	3	39.440	<0.0001***
	Water treatment*larval density	3	31.839	<0.0001***
	Feed*larval density	1	7.118	0.0076*
Pupal mortality	Larval density	1	5.007	0.0252*
	Feed	1	2.049	0.1523^ns^
	Water treatment	3	76.424	<0.0001***
	Water treatment*feed	3	42.522	<0.0001***
	Water treatment*larval density	3	8.433	0.0379*
	Feed*larval density	1	7.885	0.0050*
Adult emergence	Larval density	1	544.058	<0.0001***
	Feed	1	187.584	<0.0001***
	Water treatment	3	258.443	<0.0001***
	Feed*larval density	1	4.801	0.0285*
	Water treatment*feed	3	44.096	<0.0001***
	Water treatment*larval density	3	46.674	<0.0001***

*P*-value: ***<0.0001 (most significant), **<0.005, *<0.05, ^ns^>0.05 (not significant)

### Pupal mortality, larval density, feed types and water treatments

Overall, water treatments significantly impacted pupal mortality (Table 2). Pairwise comparisons revealed that pupal mortality was highest in NC (odds ratio test: *P* values<0.0456 in all cases) (Fig. 3b; Additional file 2: Table S2). There was no significant difference in pupal mortality between NCZ and WC (*P*=0.1382). Larval rearing density negatively impacted pupal mortality resulting in significantly higher mortality at 200 rearing density than 400 (Table 2; Fig. 3b; Additional file 2: Table S2). A significant interaction between feed and water treatment resulted in lower pupal mortality in the water change groups (WC and WCZ) than NC, but not NCZ, for slurry feed (Table 2; Fig. 3b; Additional file 2: Table S2). There was also a significant interaction between feed and density, which resulted in lower mortality at 200 rearing density for powder feed and 400 density for slurry feed (Table 2; Fig. 3b; Additional file 2: Table S2).

### Adult emergence of *An. coluzzii* across water treatment feed regimes and larval densities

Adult emergence was significantly impacted by water treatments (Table 2). Pairwise comparisons revealed that adult emergence was significantly higher in WC compared to other water treatment groups (odds ratio test: *P* values from<0.0001 in all comparisons) (Fig. 3c; Additional file 2: Table S2). Rearing density significantly impacted adult emergence, with a higher emergence rate (60%) at 200 rearing density, compared to the 43% adult emergence at 400 larval rearing density (Table 2; Fig. 3c). The significant impact of feed regime on adult emergence resulted in 10% more adults emerging from powder feed than slurry feed (Table 2). The significant interaction between water treatment and larval density resulted in differences in emergence rate among water treatment groups, with the lowest rates in 400 rearing density from NCZ (36%) and WCZ (39%) (Table 2; Fig. 3c; Table 5; Additional file 2: Table S2). The significant interaction between water treatment and feed regime resulted in the lowest emergence rates for slurry feed in NC and powder feed in NCZ (Fig. 3c; Table 2; Additional file 2: Table S2). Finally, the interaction between larval density and feed significantly impacted adult emergence resulting in 20% and 16% more adult emergence in slurry and powder feed at 200 rearing density than in 400 (Fig. 3c; Table 2; Additional file 2: Table S2).

### Mosquito survival by sex across water treatments and larval densities

Water treatment, rearing densities and feed type had no significant impact on the sex ratio of adult mosquitoes (Table 3). The sex ratio of surviving mosquitoes did not significantly deviate from the expected 50:50 ratio except at WCZ/400 larval density/powder feed (chi-square likelihood ratio test: LR=6.6728, DF=1, *P*=0.0098) and NCZ/400 larval density/powder feed (LR=5.8726, DF=1, *P*=0.0154) where females survived significantly more than males.

**Table 3:** Nominal logistic regression of mosquito survival by sex

Source	DF	Likelihood ratio	*P*-value
Water treatment	3	0.470	0.9255^ns^
Feed	1	3.001	0.0832^ns^
Larval density	1	0.141	0.7075^ns^

*P*-value: ***<0.0001 (most significant), **<0.005, *<0.05, ^ns^>0.05 (not significant)

### Effect of water treatments, feed regimes and larval density on adult wing length

**Fig. 4:**
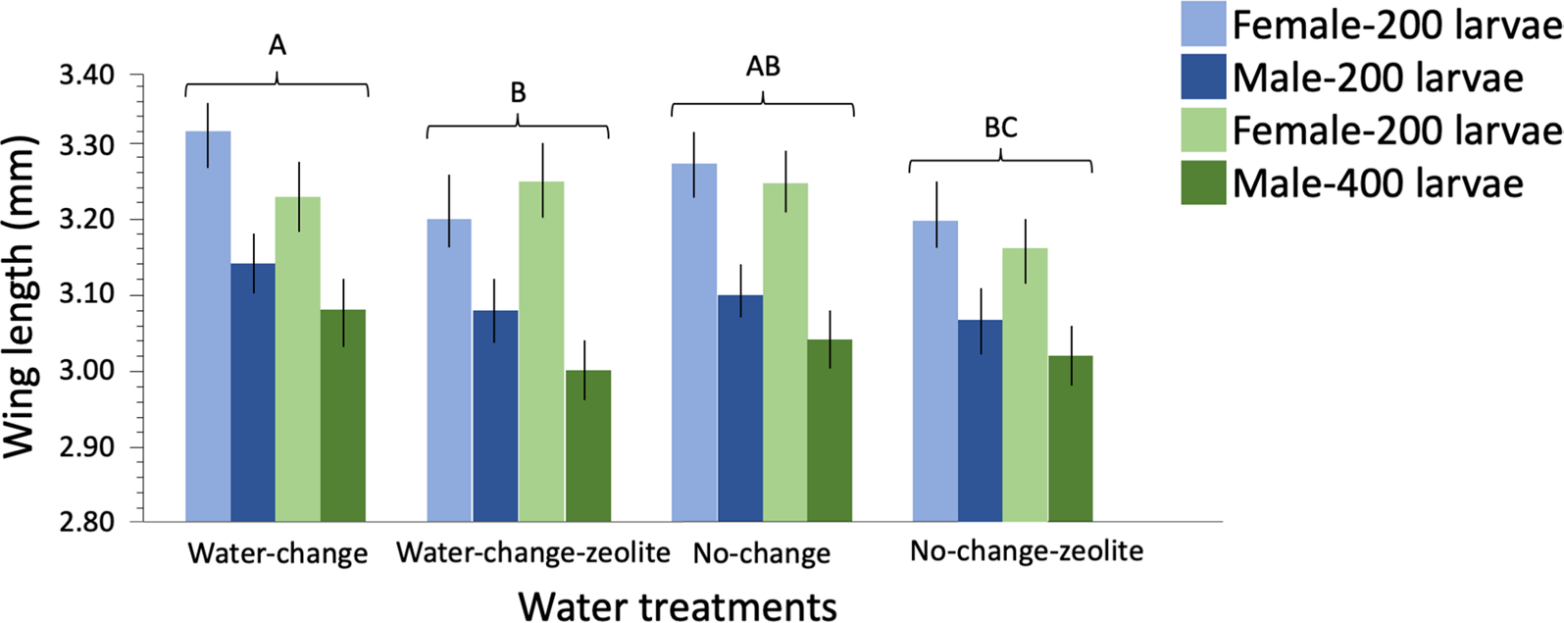
Mean wing length of emerged mosquitoes across water treatments for two larval rearing densities. Whiskers represent 95% confidence intervals (CI). Significant differences among water treatments are represented by different letters

Mosquito adult wing length was significantly impacted by water treatment (Table 4)*.* Pairwise comparisons revealed significantly longer wing length in WC than WCZ and NCZ (Tukeys HSD tests: *t*-ratios>3.62; *P* values<0.0020 in all comparisons). No significant difference in wing length was observed between WC and NC (*t*-ratios>1.36; *P*<0.5479 in all comparisons) (Fig. 4; Additional file 3: Table S3). Larval rearing density significantly impacted emerging adults' wing length, with longer wing length in 200 density than 400 (Table 4). The significant interaction between feed and water treatment resulted in longer wing length in NC, WC and WCZ for slurry than powder feed and in NCZ for powder compared to slurry feed regime (Fig. 4; Table 4). Adult wing length significantly differed by sex; females had significantly longer wing length than males (Table 4; Fig. 4; Additional file 3: Table S3).

**Table 4:** General linear model of the effect of water treatments, feed regimes and larval density on wing length

Parameter	Source	df	*F*-ratio	*P*-value
Wing length	Larval density	1	17.106	<0.0001***
	Feed	1	1.973	0.1603^ns^
	Water treatment	3	9.852	<0.0001***
	Sex	1	232.853	<0.0001***
	Water treatment*feed	3	2.998	0.0298*

*P*-value: ***<0.0001 (most significant), **<0.005, *<0.05, ^ns^>0.05 (not significant)

*df* Degrees of freedom

### Impact of larval density, water treatment and feed regimes on development time

**Fig. 5:**
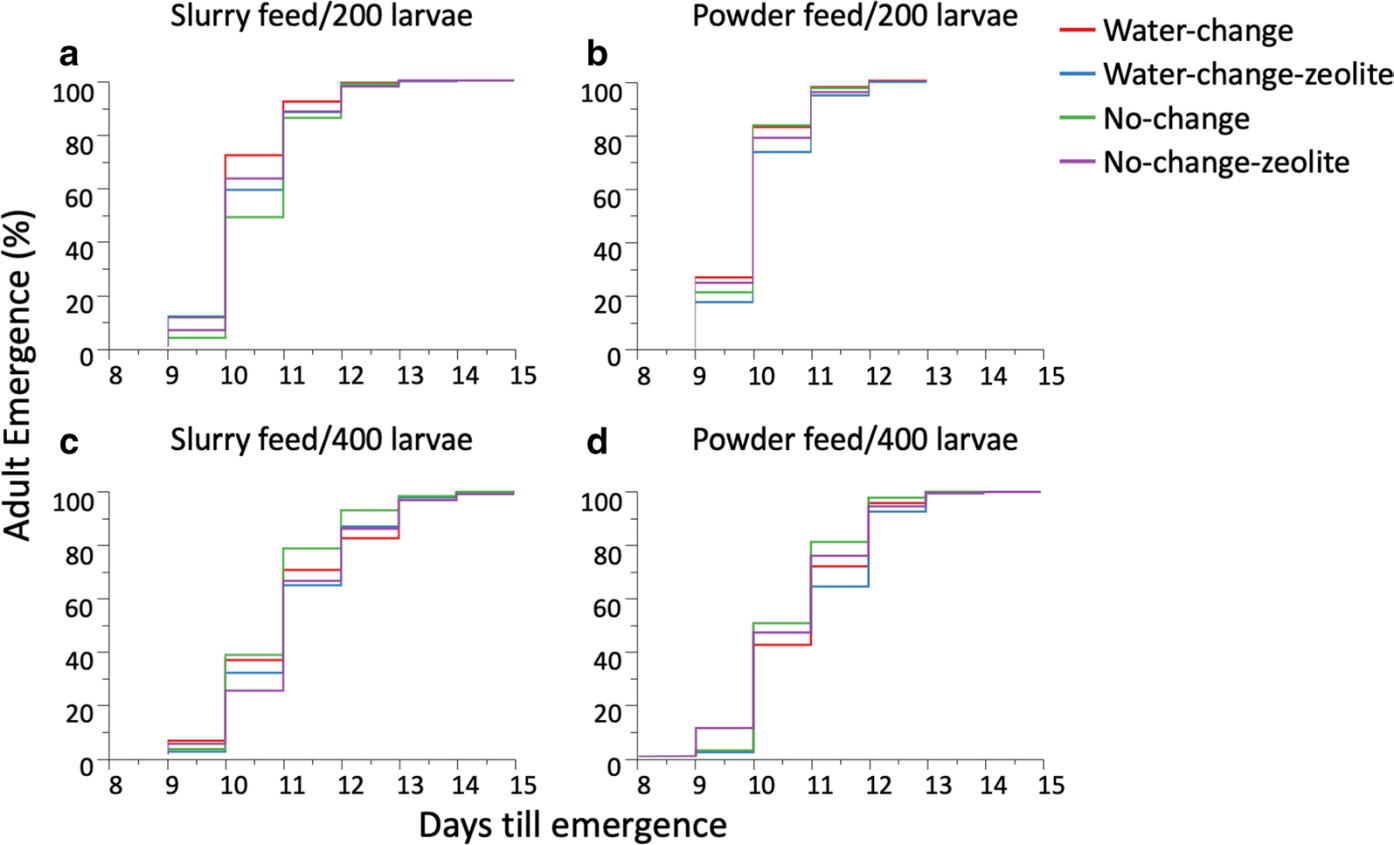
Survival curves of mosquito larvae in water treatment types by larval densities and feed regimes: **a** 200 larvae/slurry feed; **b** 200 larvae/powder feed; **c** 400 larvae/slurry feed; **d** 400 larvae/powder feed

The duration of development from first instar larvae until adult emergence (development time) was significantly impacted by water treatment (Table 5). Pairwise comparisons revealed that development time was significantly longer in WCZ compared to WC and NC (risk ratio tests: *P*0.0007 in both cases) but not compared to NCZ (*P*=0.0671) (Fig. 5; Additional file 4: Table S4). Larval rearing density significantly impacted development time, which was 1 day longer in the 400 compared to 200 rearing density (Table 5; Fig. 5; Additional file 4: Table S4). Development time was also significantly impacted by feed regimes, with mosquitoes taking more prolonged time (half-day) to complete development in slurry feed than powder feed (Table 5; Fig. 5; Additional file 4: Table S4). Significant interactions between water treatment and density resulted in the shortest development time at the 200 rearing density in WC; development time was shorter in NCZ than NC. At 400 rearing density, the shortest development time occurred in the NC water treatment (Table 5; Fig. 5; Additional file 4: Table S4). Additionally, the significant interaction between water treatment and feed regimes resulted in longer development time in NCZ than NC for mosquitoes fed with slurry feed and longer in NC than NCZ for powder feed (Table 5; Fig. 5; Additional file 4: Table S4).

**Table 5:** Cox proportional hazard analyses of the effect of water treatments, feed regimes and larval density on development time

Parameter	Source	df	Chi-square	*P*-value
Day of emergence	Larval density	1	614.460	<0.0001***
	Feed	1	142.292	<0.0001***
	Water treatment	3	18.179	0.0004**
	Water treatment*feed	3	8.365	0.0390*
	Water treatment*larval density	3	21.040	<0.0001***
	Feed*larval density	1	14.973	0.0001**

*P*-value: ***<0.0001 (most significant), **<0.005, *<0.05, ^ns^>0.05 (not significant)

## Discussion

As expected, mosquitoes reared in the trays where water was continuously refreshed provided a better larval environment for optimal mosquito growth and development. Consistently lower ammonia concentrations and higher nitrate concentrations in these trays indicated efficient conversion of toxic ammonia to nitrate [24]. Mosquito survival and adult body size were maximised in groups where water was continuously refreshed because of the absence or minimal presence of toxic compounds such as ammonia [25]. Nitrogenous wastes are known to be poisonous to aquatic organisms above certain concentrations. Previous studies have shown that ammonia negatively affects *Anopheles gambiae* (*s.l.*) development from 1.3 mg/l and that no larval development was possible at 62.5 mg/l and above [26, 27]. Here, in larval trays without water replacement, ammonia concentrations increased steadily from the 4th day and reached a peak on the 10th day. Zeolite added to the NCZ water treatment significantly decreased ammonia concentrations compared to NC trays where zeolite was not applied. Similarly, nitrate concentrations were higher from day 4 in NCZ than NC, indicating greater ammonia conversion to the less toxic nitrate [28]. The cause of overall higher mortality in *Anopheles* larval trays without water change (NC and NCZ) in comparison to those with water change (WC and WCZ) could range from hypoxia, ammonia toxicity, inability to transport oxygen, pathogenicity, nutrient enrichment and competition for food resource [29,32]. In addition, the bacterial build-up that typically accompanies waste accumulation could compound these effects by increasing ammonia production and/or potential direct bacterial toxicity [33,36].

Although not observed for overall mosquito survival, the impact of ammonia-absorbing zeolite in improving water quality in larval trays without water change was evident at the 200 larval rearing density. Adult emergence was significantly higher in NCZ than NC at the 200 larval density, thus validating zeolites ability to improve water quality in an aquaculture system based on small larval rearing trays [14, 15]. However, at higher larval density (400), the effect of zeolite was not evident for mosquito adult emergence, possibly due to two factors. First, zeolite saturation as ammonia concentration produced in the 400-larval-density trays was higher than at 200. The overcrowded trays (400 larval density) resulted in the production of relatively more elevated amounts of toxic ammonia due to the increased metabolism and waste production. Reports from the use of fish and crustacean aquaculture revealed that the greater the concentration of initial ammonia, the less the ammonia removal efficiency, providing a possible explanation for the reduced effect of ammonia adsorption by zeolite in these trays since the same amount of zeolite was used at both rearing densities [15, 20, 37].

A second but not exclusive explanation for the lack of zeolite's impact at higher density may be that ammonia reduction benefits were obscured by intra-specific competition for food and space [38]. Here, starvation resulting from intra-instar competition may have accounted for the reduced survival in trays with 400 larvae [39, 40]. Larval overcrowding is relatively common in insectaries due to lack of space and/or standardised rearing protocols, leading to suboptimal emergence rates and phenotypic quality [38, 41]. Our results suggest that zeolite might allow for rearing at higher larval densities but require higher doses of zeolites. Based on our findings we would recommend using a slightly higher amount of zeolite, i.e. 1 g per 100 larvae. However, further studies are needed to optimise the timing and dosage of zeolite water treatment to maximise its beneficial impact at different larval densities.

Zeolite water treatment also favourably impacted the duration of mosquito development time. Development time was not significantly longer in NCZ compared to the more effective continuous change WC group. This allowance for synchronous hatching and pupation using zeolite is ideal for smaller insectaries and mass-rearing facilities [42]. Any additive that can shorten pre-imaginal development time is welcome as it will reduce labour costs and enhance the accelerated production of adults [38]. This is particularly desirable in the mass rearing of adult mosquitoes for vector control/research programmes where efficient rearing systems which balance larval density, nutrition and water quality are needed [38, 43].

A crucial factor to consider for applying zeolite to improve water quality for mosquito production without water replacement is that zeolites can significantly influence the abundance and development of nitrifying microorganisms [34,36]. Additionally, un-ionised ammonia can inhibit the action of nitrifying bacteria, resulting in increased ammonia levels in aquatic habitats, thereby intensifying the harmful effects on aquatic animals and beneficial bacteria [31]. In this study, the use of zeolite prevented these ammonia spikes hence reducing any adverse carry-over effects. There is a need to understand the complex interactions between zeolite use and bacterial communities dynamics in these mosquito larval trays. For example, there was surprisingly little difference in the effect of feed and larval density on ammonia content in mosquito larval trays in this study. This is likely due to the population of nitrifying bacteria in the larval trays adjusting to feeding and larval density and increasing the conversion of ammonia to nitrates [20, 33, 36]. In that regard, powder feed was found to be better than slurry feed for mosquito development and phenotypic quality for all water treatment types. This is likely due to the greater ammonia conversion in the powder feed trays indicated by higher nitrate concentrations [28]. This may not be significant for facilities that use continuous flow systems and slurry feed, but it will be for smaller insectaries that do not conduct daily water changes [10, 13].

Overall, the higher developmental success in NCZ compared to NC (at the 200 larval rearing density) and similar phenotypic quality in NCZ compared to WC showed zeolite could be beneficial for mosquito mass-rearing. Zeolite can be particularly useful to prevent ammonia accumulation in medium- or small-scale rearing facilities constrained by space or water, allowing the rearing of anopheline mosquitoes at higher densities. This may be relevant to the often overcrowded insectaries of smaller research institutions and infrastructures in malaria-endemic countries with low GDIs (gross domestic income) in arid regions [4, 44, 45].

Surveys considering water accessibility and affordability in sub-Saharan countries show that 43% of urban households have access to piped water. In rural settings, household piped water coverage is only 4% and more expensive [46,49]. The cost of piped water in African countries typically ranges from $0.49 to $2.67/m^3^, not including connection costs and monthly fees [48, 50, 51]. In settings where water has to be supplied through bore-holes, delivery service and vendors, water costs may be fourfold higher [48, 52]. Using this study as an example, if 2 g of zeolite was applied to trays containing 1 l of water and 200 larvae, at an average cost of $0.125/kg, only $12.5 worth of zeolite would be needed to improve the water quality and production of over 10 million mosquitoes [53]. The equivalent water cost for rearing 10 million mosquitoes in sub-Saharan countries would range from $24.5 to $133.5, and our results suggest that fewer water replenishments would be needed if zeolite were used to maintain rearing water quality [4, 10, 48].

Currently, there is a dearth of literature on water management systems and water recycling and conservation in larger mosquito-rearing infrastructures [9]. In contrast to that, zeolite applications are common in closed-system fish aquaculture, which uses more water and is more advanced regarding water treatment and reuse. In future, larger mosquito production facilities might benefit from similar zeolite applications, particularly those that can decrease their reliance on freshwater and generally improve sustainability [9]. In addition to adding zeolite to rearing trays, it can be used as media in biofiltration systems where it is both cheaper and more effective than activated carbon and sandbeds and reduces both operation and maintenance costs [15, 20, 37, 54, 55]. In water systems aiming for a high proportion of water recycling, zeolite, combined with biological filters, prevents the accumulation of nitrates and may eliminate the need for denitrification chambers [56, 57]. Where more expensive RO or UF is employed, pre-filtration with zeolite commonly prevents organic build-up and membrane fouling, thereby decreasing maintenance costs [57, 58]. Following saturation, zeolitic materials are recharged by soaking in a 10% NaCl solution, thus renewing their capacity and subsequently reused [17, 18, 59]. Alternatively, the ammonia-saturated zeolite media can be used as organic fertiliser, serving as an environmentally useful by-product [15, 54]. These different examples suggest that zeolite has many possible applications for mosquito rearing facilities and may be particularly cost-effective in settings where water is expensive or difficult to access.

## Conclusions

In this study, under the no-water-change condition, zeolite reduced ammonia build-up and resulted in improved larval, pupal and adult mosquito survival as well as development time. However, this effect was not observed under all water treatment conditions suggesting that further optimisation would be required for broader applications in mosquito-rearing. Zeolite can potentially be integrated into various water management scenarios for small-to-medium- and large-scale rearing facilities to improve water quality and reduce costs. The results of this first application of zeolite water treatment to anopheline rearing are auspicious; further studies are needed to optimise zeolite dosage in relation to larval density and feed type, especially when targeting mass rearing. Similarly, a better understanding of the complex interactive effects among the use of zeolite, ammonia fluctuations and the population dynamics of beneficial and detrimental bacteria is needed to fully understand the potential benefits of zeolite and other additives for anopheline mosquito mass production.

## Supplementary Information

**Additional file 1: Table S1.** Mean nitrate and ammonia values in larval trays.

**Additional file 2: Table S2.** Mosquito survival at life history stages across water treatments.

**Additional file 3: Table S3.** Mean wing length of surviving adult *An. coluzzii* across water treatments.

**Additional file 4: Table S4.** Mean development time of *An. coluzzii* across water treatments.

## Data Availability

All datasets generated and/or analysed during this study are included in this published article and its additional files.
