# Supplementary material for: Improvement of water quality for mass anopheline rearing: evaluation of the impact of ammonia-capturing zeolite on larval development and adult phenotypic quality
Source: Parasit Vectors. 2021 May 20;14:268. doi: 10.1186/s13071-021-04763-w (PMC8139152; doi:10.1186/s13071-021-04763-w)
Supplement: Supplementary file 1 — Additional file 1: Table S1. Mean nitrate and ammonia values in larval trays. [file 13071_2021_4763_MOESM1_ESM.pdf]

Water treatment	Larval density	Feed	Day	Nitrate (NO_3^-) (mg/L)	Ammonia (NH_3) (mg/L)
WC	200	Slurry	4	10.00 (-1.25 – 21.25)	1.38 (-0.69 – 3.44)
			6	8.75 (4.77 – 12.73)	2.13 (0.87 – 3.38)
			8	10.00 (-2.99 – 22.99)	4.4 (3.49 – 5.30)
			10	0	-
		Powder	4	10.00 (-1.25 – 21.25)	1.45 (-0.08 – 2.98)
			6	26.25 (-0.83 – 53.33)	2.45 (-0.65 – 5.55)
			8	40.00 (-5.01 – 85.01)	3.65 (0.36 – 6.94)
			10	0	0.30 (-3.51 – 4.11) *2*
	400	Slurry	4	10.00 (-1.25 – 21.25)	1.45 (0.11 – 2.79)
			6	8.75 (4.77 – 12.73)	2.65 (1.60 – 3.70)
			8	32.50 (-18.44 – 83.44)	3.13 (1.00 – 5.25)
			10	2.50 (-2.09 – 7.09)	1.10 (0.12 – 2.08)
		Powder	4	10.00 (-1.25 – 21.25)	1.43 (0.08 – 2.77)
			6	45.00 (4.96 – 85.04)	2.58 (1.20 – 3.95)
			8	42.50 (-3.20 – 88.20)	3.35 (0.54 – 6.16)
			10	3.75 (-3.87 – 11.37)	1.45 (0.73 – 2.17)
WCZ	200	Slurry	4	18.75 (-5.88 – 43.38)	1.23 (-0.54 – 2.99)
			6	8.75 (4.77 – 12.73)	1.1 (-0.88 – 3.08)
			8	12.50 (4.54 – 20.46)	3.25 (2.08 – 4.42)
			10	0	0.1 (-0.33 – 0.53) *3*
		Powder	4	16.25 (-9.22 – 41.72)	1.03 (-0.30 – 2.35)
			6	35.00 (19.09 – 50.91)	1.38 (-0.22 – 2.97)
			8	60.00 (23.25 – 96.75)	3.40 (0.94 – 5.86)
			10	5.00 (-7.42 – 17.42)	0.23 (-0.77 – 1.24) *3*
	400	Slurry	4	17.50 (-8.89 – 43.89)	0.45 (-0.58 – 1.48)
			6	20.00 (-2.50 – 42.50)	0.75 (-0.16 – 1.66)
			8	16.25 (-9.22 – 41.72)	3.68 (2.55 – 4.80)
			10	1.25 (-2.73 – 5.23)	0.40 (-0.37 – 1.17)
		Powder	4	26.25 (-0.83 – 53.33)	1.05 (0.31 – 1.79)
			6	45.00 (4.96 – 85.04)	1.28 (0.48 – 2.07)

Water treatment	Larval density	Feed	Day	Nitrate (NO_3^-) (mg/L)	Ammonia (NH_3) (mg/L)
			8	60.00 (23.25 – 96.75)	3.45 (1.27 – 5.63)
			10	25.00 (-35.25 – 85.24)	0.57 (-1.26 – 2.40) *3*
NC	200	Slurry	4	18.75 (-5.88 – 43.38)	1.98 (-0.57 – 4.52)
			6	2.50 (-2.09 – 7.09)	4.25 (2.65 – 5.85)
			8	0	13.85 (10.92 – 16.78)
			10	0	12.57 (2.46 – 22.67) *3*
		Powder	4	13.75 (-14.84 – 42.34)	1.13 (-0.68 – 2.93)
			6	27.5 (3.63 – 51.37)	4.25 (1.84 – 6.66)
			8	7.50 (-6.28 – 21.28)	12.43 (6.26 – 18.59)
			10	0	12.27 (7.79 – 16.75)
	400	Slurry	4	26.25 (-0.83 – 53.34)	0.73 (-0.13 – 1.58)
			6	7.50 (-0.46 – 15.46)	5.23 (0.27 – 10.18)
			8	1.25 (-2.73 – 5.23)	12.70 (12.01 – 13.39)
			10	0	15.38 (4.82 – 25.93)
		Powder	4	16.25 (-9.22 – 41.72)	1.08 (0.28 – 1.87)
			6	36.25 (-15.46 – 87.96)	6.20 (0.64 – 11.76)
			8	35.00 (-19.35 – 89.35)	13.91 (11.37 – 16.44)
			10	0	15.43 (4.66 – 26.19)
NCZ	200	Slurry	4	18.75 (-5.88 – 43.38)	1.6 (-0.31 – 3.51)
			6	18.75 (-5.88 – 43.38)	2.25 (0.47 – 4.03)
			8	2.50 (-5.46 – 10.46)	8.53 (5.73 – 11.32)
			10	0	6.73 (0.42 – 13.05) *3*
		Powder	4	11.25 (1.24 – 21.26)	1.03 (-0.24 – 2.29)
			6	35.00 (19.09 – 50.91)	2.28 (0.05 – 4.50)
			8	15.00 (-11.78 – 41.78)	7.80 (5.94 – 9.66)
			10	0	8.03 (6.34 – 9.72) *3*
	400	Slurry	4	26.25 (-0.83 – 53.33)	1.28 (-0.47 – 3.02)
			6	6.25 (-1.37 – 13.87)	2.28 (-1.66 – 6.21)
			8	0	8.13 (5.46 – 10.79)
			10	0	8.55 (-2.54 – 19.64)
		Powder	4	23.75 (-6.28 – 53.78)	1.13 (-0.05 – 2.30)

Water treatment	Larval density	Feed	Day	Nitrate (NO_3^-) (mg/L)	Ammonia (NH_3) (mg/L)
			6	51.25 (-6.26 – 108.76)	2.60 (-0.34 – 5.54)
			8	25.00 (-35.24 – 85.24)	8.05 (6.16 – 9.94)
			10	0	10.45 (0.94 – 19.96)

Notes: Sample size is 4 except in cases where pupation is completed in trays, then italicised. Ninety-five percent confidence intervals are in brackets.
