# Supplementary material for: Improvement of water quality for mass anopheline rearing: evaluation of the impact of ammonia-capturing zeolite on larval development and adult phenotypic quality
Source: Parasit Vectors. 2021 May 20;14:268. doi: 10.1186/s13071-021-04763-w (PMC8139152; doi:10.1186/s13071-021-04763-w)
Supplement: Supplementary file 2 — Additional file 2: Table S2. Mosquito survival at life history stages across water treatments. [file 13071_2021_4763_MOESM2_ESM.pdf]

Feed	Larval density	Treatment	%Larval survival	%Pupal mortality	%Adult emergence
Slurry	200	WC	75 (71-77)	7 (5-9)	68 (65-71)
		WCZ	62 (58-65)	8 (6-10)	54 (51-58)
		NC	61 (57-64)	16 (13-18)	45 (42-49)
		NCZ	62 (59-65)	6 (4-8)	56 (53-60)
Powder	200	WC	79 (76-82)	10 (8-12)	69 (66-73)
		WCZ	73 (70-76)	6 (4-8)	67 (64-70)
		NC	66 (63-69)	8 (6-10)	58 (54-61)
		NCZ	72 (69-75)	9 (7-11)	63 (60-67)
Slurry	400	WC	53 (51-56)	4 (3-5)	49 (47-52)
		WCZ	34 (32-36)	4 (4-6)	30 (27-32)
		NC	44 (42-46)	10 (9-12)	34 (31-36)
		NCZ	43 (41-45)	9 (8-11)	34 (32-36)
Powder	400	WC	69 (66-71)	9 (8-11)	60 (57-62)
		WCZ	55 (52-57)	6 (5-7)	49 (46-51)
		NC	58 (55-60)	10 (9-12)	48 (45-50)
		NCZ	46 (44-49)	8 (7-9)	38 (36-41)

Ninety-five percent confidence intervals are in brackets. Larval survival, pupal mortality and adult emergence were calculated out of the initial sample sizes of 800 (200 larval density) and 1600 (400 larval density).
