# Supplementary material for: Improvement of water quality for mass anopheline rearing: evaluation of the impact of ammonia-capturing zeolite on larval development and adult phenotypic quality
Source: Parasit Vectors. 2021 May 20;14:268. doi: 10.1186/s13071-021-04763-w (PMC8139152; doi:10.1186/s13071-021-04763-w)
Supplement: Supplementary file 3 — Additional file 3: Table S3. Mean wing length of surviving adult An. coluzzii across water treatments. [file 13071_2021_4763_MOESM3_ESM.pdf]

Water Treatment	Larval density	Sex	Mean wing length (mm)
WC	200	Female	3.31 (3.26 – 3.35)
		Male	3.15 (3.11– 3.18)
	400	Female	3.23 (3.18 –3.27)
		Male	3.08 (3.03 – 3.12)
WCZ	200	Female	3.20 (3.16 – 3.25)
		Male	3.08 (3.04 – 3.12)
	400	Female	3.25 (3.20 – 3.30)
		Male	3.00 (2.95 – 3.04)
NC	200	Female	3.27 (3.23 – 3.32)
		Male	3.10 (3.07 – 3.14)
	400	Female	3.25 (3.21 – 3.29)
		Male	3.05 (3.01 – 3.08)
NCZ	200	Female	3.20 (3.15 – 3.25)
		Male	3.07 (3.03 – 3.11)
	400	Female	3.16 (3.11 - 3.20)
		Male	3.02 (2.98 – 3.06)

Notes: Ninety-five percent confidence intervals are in parentheses and the sample row is 80.
