# Supplementary material for: Improvement of water quality for mass anopheline rearing: evaluation of the impact of ammonia-capturing zeolite on larval development and adult phenotypic quality
Source: Parasit Vectors. 2021 May 20;14:268. doi: 10.1186/s13071-021-04763-w (PMC8139152; doi:10.1186/s13071-021-04763-w)
Supplement: Supplementary file 4 — Additional file 4: Table S4. Mean development time of An. coluzzii across water treatments. [file 13071_2021_4763_MOESM4_ESM.pdf]

Treatment	Larval density	Feed	Days till emergence
WC	200	Slurry	10.27 (10.20 – 10.34) *543*
		Powder	9.95 (9.89 – 10.01) *558*
	400	Slurry	11.11 (11.02 – 11.20) *790*
		Powder	10.89 (10.83 – 10.95) *951*
WCZ	200	Slurry	10.45 (10.36 – 10.53) *433*
		Powder	10.17 (10.10 – 10.24) *537*
	400	Slurry	11.21 (11.10 – 11.31) *472*
		Powder	10.97 (10.89 – 11.05) *778*
NC	200	Slurry	10.64 (10.56 - 10.73) *361*
		Powder	10.00 (9.93 – 10.06) *462*
	400	Slurry	10.92 (10.83 – 11.00) *540*
		Powder	10.70 (10.63 – 10.76) *765*
NCZ	200	Slurry	10.46 (10.38 – 10.55) *450*
		Powder	10.02 (9.95 – 10.09) *506*
	400	Slurry	11.25 (11.15 – 11.35) *542*
		Powder	10.74 (10.65 – 10.83) *614*

Notes: Ninety-five percent confidence intervals are in parentheses and the sample sizes are italicised.
